# On the use of continuous flash suppression for the study of visual processing outside of awareness

**DOI:** 10.3389/fpsyg.2014.00724

**Published:** 2014-07-11

**Authors:** Eunice Yang, Jan Brascamp, Min-Suk Kang, Randolph Blake

**Affiliations:** ^1^School of Optometry, University of California at BerkeleyBerkeley, CA, USA; ^2^Helmholtz Institute and Division of Experimental Psychology, Department of Psychology, Utrecht UniversityUtrecht, Netherlands; ^3^Department of Psychology, Sungkyunkwan UniversitySeoul, Republic of Korea; ^4^Center for Neuroscience Imaging Research, Institute for Basic ScienceDaejeon, Republic of Korea; ^5^Department of Psychology, Vanderbilt UniversityNashville, TN, USA; ^6^Department of Brain and Cognitive Sciences, Seoul National UniversitySeoul, Republic of Korea

**Keywords:** continuous flash suppression, binocular rivalry, interocular suppression, unconscious processing, visual processing

## Abstract

The interocular suppression technique termed continuous flash suppression (CFS) has become an immensely popular tool for investigating visual processing outside of awareness. The emerging picture from studies using CFS is that extensive processing of a visual stimulus, including its semantic and affective content, occurs despite suppression from awareness of that stimulus by CFS. However, the current implementation of CFS in many studies examining processing outside of awareness has several drawbacks that may be improved upon for future studies using CFS. In this paper, we address some of those shortcomings, particularly ones that affect the assessment of unawareness during CFS, and ones to do with the use of “visible” conditions that are often included as a comparison to a CFS condition. We also discuss potential biases in stimulus processing as a result of spatial attention and feature-selective suppression. We suggest practical guidelines that minimize the effects of those limitations in using CFS to study visual processing outside of awareness.

## INTRODUCTION

During our waking hours our eyes provide us with more sensory information than we can possibly process in detail, and only a small proportion of this information reaches awareness. At the same time, it would be adaptive for our brains to continue monitoring potentially relevant sensory signals, even those that do not culminate in a conscious experience. Indeed, several lines of research suggest that unperceived visual information can influence perceptual and cognitive operations, without our awareness (reviews by Bridgeman, 1992; Merikle and Daneman, 1998; Goodale and Milner, 2004).

While the notion of processing outside of awareness^1^ is intriguing, it remains one of the most controversial issues in psychology, and for decades the research area has been fraught with methodological and theoretical challenges (e.g., Eriksen, 1960; Marcel, 1983; Holender, 1986; Merikle and Daneman, 1998). Yet at the same time, psychophysical techniques for rendering stimuli perceptually invisible continue to be developed, providing researchers with an ever more varied array of experimental tools for investigating processing outside of awareness (review by Kim and Blake, 2005). Some of these tools exploit the reflexive suppression that occurs when different images are simultaneously presented to the two eyes, i.e., dichoptic stimulation. An advantage of dichoptic stimulation techniques over other approaches is that an observer can monocularly view one of any variety of salient stimuli, yet remain unaware of its presence for seconds at a time. Variants of this dichoptic stimulation technique include binocular rivalry (Wheatstone, 1838; Breese, 1909), flash suppression (Wolfe, 1984), generalized flash suppression (Wilke et al., 2003), flicker-swap rivalry (Logothetis et al., 1996), and binocular switch suppression (Arnold et al., 2008). One version that has recently become popular as a means for erasing visual stimuli from awareness is called continuous flash suppression (CFS; Tsuchiya and Koch, 2005), and it is the focus of our paper.

While traditional binocular rivalry typically involves two displays of roughly similar “potency” (e.g., comparable motion content and luminance contrast) being presented to the two eyes, CFS critically involves a much less balanced design. During CFS one eye views rapidly flashing contour-rich patterns of high contrast (sometimes referred to as dynamic Mondrians), while the other views a stimulus that is typically stationary and of moderate contrast. The ever-changing patterns viewed by one eye cause periods of invisibility of the unchanging stimulus viewed by the other eye, and these periods can last for dozens of seconds, about 10 times longer than suppression produced with traditional binocular rivalry (Tsuchiya and Koch, 2005). CFS has several attractive features. For instance, anecdotal observations by several laboratories indicate that the suppressive effect of CFS can engulf even relatively large stimuli presented to the other eye, stimuli that yield pronounced piecemeal suppression when viewed during binocular rivalry (e.g., Meenes, 1930; Blake et al., 1992). In addition, with CFS complete invisibility can reliably be induced from the very onset of stimulus presentation, in contrast to the situation during traditional binocular rivalry where the initially suppressed stimulus can be unpredictable (e.g., Carter and Cavanagh, 2007; Song and Yao, 2009) and subsequent fluctuations in suppression transpire unpredictably between the two rival stimuli (e.g., Fox and Herrmann, 1967). While masking and attentional blink paradigms, like CFS, allow control over the onset timing of invisibility, those two techniques are constrained by allowing only very brief stimulus durations (Kim and Blake, 2005). Furthermore, in comparison to paradigms like crowding and motion induced blindness, perceptual suppression with CFS is less susceptible to the effects of unstable fixation and eye movements (Kim and Blake, 2005). Given these properties of the perceptual suppression induced by CFS, it is not surprising that CFS has been quickly and widely adopted as a tool for investigating visual processing outside of awareness.

When looking at findings from studies using CFS, the evidence for stimulus processing outside of awareness seems compelling. As reviewed below, CFS suppression does not appear to preclude neural processing, either of low-level stimulus features, or of abstract stimulus properties with dedicated representations at more advanced stages of the visual system. In the case of low-level features, the notion that these are registered outside of awareness has also been confirmed using a number of other perceptual suppression techniques, including traditional binocular rivalry. With regard to more advanced stages of analysis, however, the picture from the literature as a whole is worth revisiting, given that there is little evidence for high-level processing during suppression phases of traditional binocular rivalry which, ironically, is reputed to create weaker interocular suppression than does CFS (Tsuchiya and Koch, 2005;Tsuchiya et al., 2006).

In addition to this apparent discrepancy between CFS and traditional binocular rivalry, another motivation behind this review is our conviction that CFS experiments into processing outside of awareness, although often straightforward in their basic idea, are surprisingly complicated, and their design and interpretation are fraught with subtleties. Careful consideration of these subtleties is particularly important because work that uses CFS for this purpose may have a significant impact on current theories of neural information processing, emotional processing, and psychopathology. Indeed, the utilization of CFS has already found its way into clinical research (Sterzer et al., 2011; Sylvers et al., 2011; Yang et al., 2011), and so it is imperative that CFS be used wisely to study processing outside of awareness.

The objective of our paper is to recommend practical guidelines for researchers interested in exploring this technique as a means of investigating stimulus processing outside of awareness. Our recommendations are centered on answering four primary questions:

(1) What are suitable paradigms to use with CFS to study processing outside of awareness?
(2) What are the optimal ways to determine whether a stimulus is genuinely invisible?
(3) What are effective methods for gaging the specificity and strength of stimulus processing outside of awareness?
(4) What are factors that influence the robustness of stimulus-driven effects under CFS?

We draw attention to several considerations that should be made when answering each of these questions. For the first question, we review the common approaches used to study processing outside of awareness with CFS. For the second question, we reexamine the methods used to assess observers’ perception during CFS suppression, since evidence for processing outside of awareness rests critically on demonstrating absence of awareness. This is particularly pertinent to behavioral priming and adaptation paradigms as well as neurophysiological studies that use CFS to examine stimulus processing outside of awareness. For the third question, we discuss the application of “visible” conditions used to provide a comparison for CFS conditions in order to determine the specificity and strength of stimulus-driven effects during CFS. For the fourth question, we discuss mechanisms that can potentially modulate stimulus processing under CFS such as those engaged in attention and feature-selective suppression. Following the discussion of each issue, we propose strategies for effectively resolving these questions and for minimizing methodological confounds in using CFS to study processing outside of awareness.

From the outset we acknowledge that the matter of “processing outside of awareness” is fraught with controversy, with sharp points of disagreement within the field. Our views are unlikely to be received without question by all who are interested in this issue. We do hope that our views can advance the conversation in a constructive way.

### WHAT ARE SUITABLE PARADIGMS TO USE WITH CFS TO STUDY PROCESSING OUTSIDE OF AWARENESS?

**FIGURE 1:**
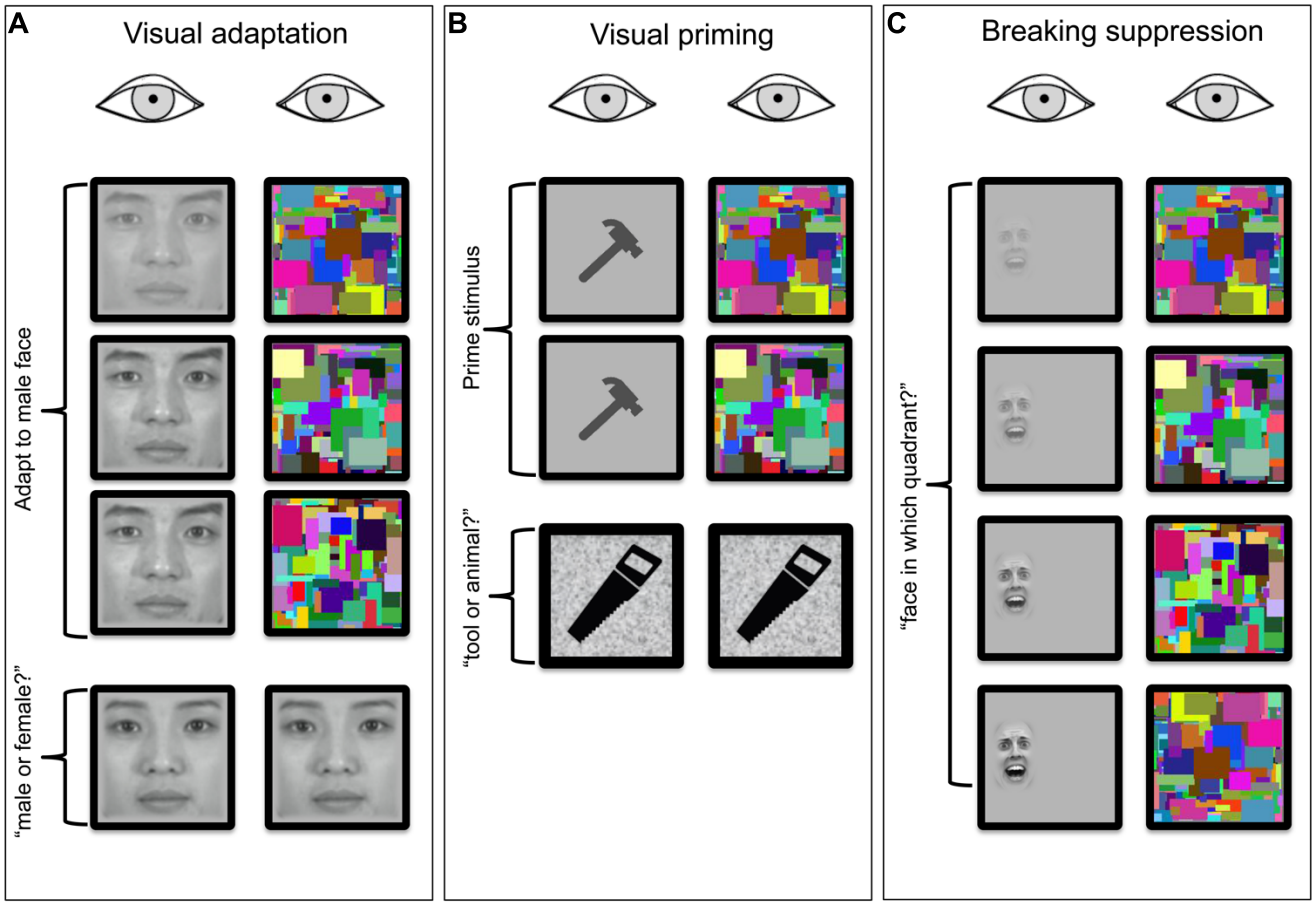
**Schematics of three frequently employed test procedures for assessing the effect of interocular suppression induced by CFS on visual processing.** In all three panels, time runs from the top to the bottom. **(A)** Visual adaptation stimulus (a male face in this example) is presented to one eye for a duration ordinarily sufficient to generate a visual aftereffect (shift in perceived gender of neutral test face viewed following adaptation). Is the aftereffect attenuated or abolished consequent to suppression of awareness of the male face by the CFS array viewed by the other eye? The adapting face is turned on gradually over several hundred milliseconds to avoid abrupt transients that can briefly perturb suppression (Photographs courtesy of Sang Chul Chong, Yonsei University). **(B)** Visual priming stimulus is briefly presented to one eye while the other eye views a CFS array. Does this prime stimulus influence accuracy and/or speed of performance on a subsequent object categorization task? **(C)** bCSF trial where a stimulus presented to one eye remains present until the observer has sufficient information to specify the location of the target (in this example a 2AFC detection task). In one variant of this b-CFS task, the observer is simply asked to indicate when the monocular stimulus achieves dominance. With b-CFS the experimenter is usually interested in learning whether provocative (e.g., fearful face) or atypical (e.g., inverted face) stimuli yield significantly different times-to-dominance compared to putatively neutral versions of the same class of stimuli (Face © Bantosh/ CC-BY-SA-3.0).

In this section, we review research that has used CFS to ask whether visual processing can transpire outside of awareness (**Figure 1**). We focus on three types of behavioral effects that have been investigated to tackle this question: (1) adaptation aftereffects of suppressed stimuli, (2) priming effects evoked by suppressed stimuli, and (3) dependence of suppression duration on the nature of the suppressed stimulus. In each case we will also briefly address how recent CFS findings compare to previous findings obtained using traditional binocular rivalry.

#### Adaptation aftereffects with CFS

Several CFS studies have utilized the well-established adaptation paradigm in which exposure to a stimulus gives rise to visual aftereffects (**Figure 1A**). A variety of different aftereffects exist, each specifically affecting detection or appearance of particular stimulus attributes, ranging from low-level properties such as orientation (e.g., the tilt aftereffect) to high-level features such as face identity and facial expression. Aftereffects have been widely used psychophysically to isolate and probe neural mechanisms involved in processing particular stimulus attributes (Mollon, 1974; Thompson and Burr, 2009). One way to investigate the extent of stimulus encoding outside of awareness is to determine whether aftereffects can be induced and, by inference, whether neural adaptation occurs, when the inducing stimulus is suppressed from awareness during the adaptation period. If full-strength adaptation aftereffects can be induced despite perceptual suppression, it stands to reason that the neural events responsible for adaptation transpire regardless of observers’ awareness of the inducing stimulus. On the other hand, these neural events may be affected by suppression, resulting in aftereffects that are weakened or even abolished (Blake et al., 2006). Considering that induction of strong aftereffects typically requires longer periods of visual adaptation, CFS – because of the enduring suppression it produces – is particularly suitable for testing adaptation aftereffects of invisible stimuli.

In some cases when this strategy was applied with CFS to suppress an adapting stimulus, CFS effectively weakened, but not necessarily abolished, the resulting adaptation aftereffect. This is true for aftereffects specific to stimulus properties often ascribed to early visual processing, including spatial phase (van Boxtel et al., 2010b), orientation (Kanai et al., 2006; Bahrami et al., 2008), motion (Maruya et al., 2008; Kaunitz et al., 2011a), and contrast (Shin et al., 2009; Yang et al., 2010b). At the same time, aftereffects thought to result from adaptation within “high-level” stages of visual processing are typically abolished entirely by suppression – examples in this category include aftereffects induced by adaptation to complex motion (Maruya et al., 2008; Kaunitz et al., 2011a), to curvature (Sweeny et al., 2011) and to faces specific for race (Amihai et al., 2011), for gender (Shin et al., 2009; Amihai et al., 2011), for gaze (Stein et al., 2012a), for face shape (Stein and Sterzer, 2011), and for emotional expression (Yang et al., 2010b; but see Adams et al., 2010). The attenuation of early visual adaptation and the complete disruption of high-level visual adaptation during CFS are fairly consistent with results obtained using the adaptation aftereffect paradigm in conjunction with traditional binocular rivalry (see Blake and He, 2005 for review).

Studies of adaptation aftereffects clearly demonstrate that CFS interferes with the neural analysis of diverse stimulus attributes. Moreover, if suppression greatly influences encoding of fundamental visual properties such as orientation and contrast, one could reasonably assume that it would similarly affect encoding of more complex image properties that are defined by combinations of these features. However, other lines of research suggest that certain classes of complex properties continue to be processed despite being blocked from visual awareness by CFS. One source of support is evidence of priming effects from stimuli suppressed with CFS, the topic we turn to next.

#### Priming effects with CFS

Subliminal priming procedures are among the most established and most popular techniques used to investigate visual processing outside of awareness (**Figure 1B**). These procedures build on traditional priming paradigms that demonstrate improved performance on tasks that involve a target stimulus, when presentation of that stimulus is preceded by presentation of a (prime) stimulus that shares physical or conceptual (semantic) characteristics of the target stimulus (Marcel, 1983; reviews by Snodgrass et al., 2004; Kouider and Dehaene, 2007). When the prime stimulus is suppressed from awareness but nonetheless engenders a priming effect on the subsequently viewed target, the presumption is that the stimulus feature or characteristic responsible for priming has been neurally registered despite the phenomenological suppression of the prime.

In various studies using CFS as the agent of suppression, invisible primes remained effective on tasks where the target was identical to the prime (Faivre et al., 2012), on tasks where the targets were semantically or categorically related to the prime (Almeida et al., 2008, 2010; Zabelina et al., 2013; but see Kang et al., 2011; Sakuraba et al., 2012), and on tasks where the targets were similar in their numerosity (Bahrami et al., 2010). To give one specific example, Almeida et al. (2008) presented images of objects drawn from different categories (i.e., animals, tools, vehicles) as prime and target stimuli, and prime stimuli were rendered invisible owing to CFS. Observers’ reaction times (RTs) in categorizing targets were reduced when these were objects in the tool category that were preceded by an invisible prime that was also a tool, but this priming effect was not found for the other object categories. This category-specific priming effect suggests that objects in the tool category may be preferentially processed without awareness. Another set of findings demonstrated that emotional expressions presented under CFS biased observers’ preference toward subsequently presented neutral stimuli (Anderson et al., 2012; Almeida et al., 2013; but see Faivre et al., 2012; de Zilva et al., 2013). These results indicate that stimuli presented outside of awareness can undergo analysis specific to relatively abstract properties like numerosity, object category and emotional content, thus leading to observable perceptual or decisional biases.

Unlike the situation for the literature on aftereffects and CFS, results on priming and CFS seem at odds with the pattern of findings reported when traditional binocular rivalry is used to manipulate awareness. In contrast to the CFS findings described above, both semantic priming effects with words and repetition priming effects with pictorial images were found to be completely abolished when prime stimuli were suppressed under binocular rivalry (Zimba and Blake, 1983; Cave et al., 1998). Considering that CFS is a stronger form of interocular suppression than binocular rivalry (Tsuchiya et al., 2006) one would, if anything, expect the effectiveness of a suppressed prime stimulus to be even weaker in the case of CFS. The source of this discrepancy between CFS and binocular rivalry priming studies has yet to be elucidated, but one factor to keep in mind pertains to the temporal buildup of suppression produced by CFS. Tsuchiya et al. (2006) found that the potency of suppression is initially relatively weak and builds up with successive mask presentations, plateauing after about 500 ms (i.e., the appearance of five successive masks). Therefore suppression may be shallow at shorter presentation durations of the CFS display, which happen to be adopted by some priming studies (Almeida et al., 2008, 2010, 2013; Sakuraba et al., 2012).

#### Emergence from suppression with CFS

The third and final line of research discussed here that utilizes CFS in investigating processing outside of awareness entails measuring the amount of time that a stimulus remains suppressed under CFS (**Figure 1C**). The assumption in these studies is that particular stimuli or categories of stimuli that emerge more quickly from suppression, relative to other stimuli, are being registered despite being suppressed owing to CFS. Unlike adaptation and priming paradigms in which stimulus awareness and behavioral effects driven by processes outside of awareness are measured independently, the “breaking continuous flash suppression” (b-CFS) technique provides a measure of stimulus awareness with which one may be able to infer processing outside of awareness. As a result of this property of b-CFS, there exists disagreement in the literature regarding the extent to which b-CFS actually provides a valid measure of unconscious processing (Stein et al., 2011b).

Breaking continuous flash suppression is based on a hallmark characteristic of binocular rivalry: stronger stimuli (e.g., high contrast stimulus) remain suppressed for shorter periods of time (Levelt, 1965). Using this technique, Jiang et al. (2007) presented either upright or inverted face stimuli to an observer’s suppressed eye while a CFS mask was presented to the other eye, and they measured the time it took for the observer to report the location of the face as it emerged from suppression. Upright faces were detected faster than inverted faces (also see Yang et al., 2007; Stein et al., 2011b), implying that upright faces were effectively stronger stimuli than inverted faces. Jiang et al. (2007) interpreted this result to mean that invisible upright faces were processed at the level of object category, given that basic stimulus features do not vary importantly with variations in face orientation whereas face recognition is highly susceptible to face orientation. This finding has inspired others to investigate processing outside of awareness of social and emotional cues of faces using the same CFS technique. These studies have found that faces with fearful expressions tend to break suppression more quickly than other facial expressions (Yang et al., 2007; Tsuchiya et al., 2009; Sterzer et al., 2011; Gray et al., 2013; Stein et al., 2014), as do faces with eyes that gaze directly at the observer (Stein et al., 2011a) and faces judged as trustworthy or as non-domineering (Stewart et al., 2012).

Breaking continuous flash suppression has also been used to examine whether other high-level properties, including lexical and semantic information, are processed outside of awareness. For instance, images of morphemes that are part of one’s native language tend to emerge from suppression faster than images of unfamiliar, foreign words (Jiang et al., 2007). Similarly, an initially suppressed word breaks suppression more quickly if that word is preceded by a semantically related visible word (Costello et al., 2009). The affective connotation of a word or phrase may also modulate the duration of suppression under CFS (Yang and Yeh, 2010; Sklar et al., 2012). Finally, the time to break from suppression is also reduced for stimuli that are semantically congruent with concurrently presented stimuli delivered through another sensory modality – this bisensory facilitation is found with hearing (Alsius and Munhall, 2013; Lupyan and Ward, 2013) and with olfaction (Zhou et al., 2010). Overall, the existing evidence implies that with b-CFS, semantic information of an invisible stimulus may be encoded and, consequently, strengthen the neural signals associated with that stimulus, empowering it to emerge more quickly from suppression.

Again we can compare these results obtained using b-CFS with those found using traditional binocular rivalry. Binocular rivalry findings are similar in showing that cognitively salient (i.e., meaningful) stimuli exhibit predominance over less meaningful stimuli (Walker, 1978; review by Blake and Logothetis, 2002). For example, recognizable figures (Yu and Blake, 1992), familiar images (Engel, 1956; Losciuto and Hartley, 1963), and emotional faces (e.g., Alpers and Gerdes, 2007; Bannerman et al., 2008) enjoy prolonged perceptual dominance. Note, however, an important difference between the measures of “time to break from suppression” during b-CFS and predominance during binocular rivalry. Because binocular rivalry involves alternating perception of both eyes’ images, changes in predominance can often be explained by altered processing of the perceptually dominant stimulus rather than any processing occurring in the suppression phase of rivalry. Indeed it is well-established that, for instance, attention to the perceptually dominant stimulus increases its dominance durations (Lack, 1978; Ooi and He, 1999; Meng and Tong, 2004; Chong et al., 2005; Hugrass and Crewther, 2012). In this sense, the time a stimulus takes to break initial suppression during b-CFS can provide a more unequivocal answer than can binocular rivalry, depending on the question being asked. It is also worth noting that binocular rivalry studies using test probe techniques often rely on stimulus discrimination (e.g., Ling and Blake, 2009) or recognition (e.g., Alais and Melcher, 2007) to gage the depth of suppression whereas b-CFS studies tend to use detection or stimulus localization. Different tasks could contribute to apparent differences in interpretation of results from rivalry and b-CFS.

#### Recommendations

Adaptation, priming, and b-CFS are all adapted from well-established techniques in studying stimulus processing. Priming and b-CFS techniques may be suitable for investigating both perceptual and higher-level cognitive processes outside of awareness, whereas visual adaptation may be optimal for examining predominantly low-level visual processes and some complex ones as well (i.e., face processing). Before deciding which technique to use, experimenters should also consider the shortcomings of the current implementation of each technique, which are discussed throughout the remaining parts of this paper.

### WHAT ARE THE OPTIMAL WAYS TO DETERMINE WHETHER A STIMULUS IS GENUINELY INVISIBLE?

As with any technique used to study subliminal perception, CFS studies that report performance or physiological measures indicative of stimulus processing outside of awareness must demonstrate that the stimuli were genuinely suppressed from awareness. In priming and adaptation paradigms, measures of awareness are assessed independently of the measure of processing outside of awareness (i.e., priming effect and aftereffect). In contrast, b-CFS provides an index of awareness to infer stimulus processing outside of awareness, and so this section does not pertain to the b-CFS paradigm. In establishing the absence of awareness, some researchers advocate subjective measures (e.g., Cheesman and Merikle, 1986) whereas others argue for the use of objective measures (e.g., Holender, 1986) to verify observers’ unawareness of stimuli suppressed by CFS.

A very popular way of obtaining a subjective measure of awareness in the context of CFS experiments is to ask participants to report any occasion when they perceive another image besides the CFS suppressor. These subjective reports are then used to discard trials where suppression fails (e.g., Kanai et al., 2006; Maruya et al., 2008). Rather than relying on binary judgments of visibility (“yes” versus “no”), there are other, more nuanced ways to cull visible from invisible trials (Sandberg et al., 2010; see Hesselmann, 2013 for review). For instance, observers can rate the quality of their visual experience on a graded scale, such as the perceptual awareness scale (PAS), which includes multiple response options ranging from “no visual experience at all” on one extreme, to “a clear and complete visual experience” on the other (Ramsoy and Overgaard, 2004; see Ludwig et al., 2013 for a CFS study using PAS). In some approaches, reports of subjective experience are supplemented by asking observers to provide confidence ratings of these reports (e.g., Cheesman and Merikle, 1986; Kunimoto et al., 2001). For instance, in one recently introduced form of confidence rating termed post-decision wagering, observers’ confidence levels are represented by the amount of money they are willing to bet on the accuracy of their subjective judgments (Persaud et al., 2007). The method should in principle motivate people both to respond in a bias-free manner and to accurately express their confidence level (Persaud et al., 2007; Schurger and Sher, 2008; but see Clifford et al., 2008). Another approach to investigating awareness using confidence ratings aims to characterize the nature of invisibility on trials where observers report seeing no stimulus, by combining confidence ratings on these trials with signal detection theory (Kanai et al., 2010). Only a few studies have applied confidence rating methods to study stimulus analysis during CFS (Bahrami et al., 2008; Sterzer et al., 2008; Raio et al., 2012).

In other published studies, a 2-alternative categorization task has been used to infer the extent to which an observer is aware of a stimulus viewed together with CFS. In two versions of this approach, observers are instructed to either classify the suppressed stimulus into one of two object categories (e.g., tool versus animal; e.g., Almeida et al., 2008; Arnold et al., 2008; Sterzer et al., 2008; Kaunitz et al., 2011b; Raio et al., 2012) or to discriminate the suppressed stimulus from a grid-scrambled version of that stimulus (Fang and He, 2005; Jiang and He, 2006; Jiang et al., 2006, 2009). Because such tasks require the observer to make a report following CFS presentation, and because many paradigms also involve another behavioral report at that time, these categorization tasks are often implemented in a separate “control” experiment rather than as part of the main experiment, to avoid dual task demands. If performance is not significantly different from chance in the control experiment, investigators conclude that observers were also unaware of the stimuli presented under CFS during the condition of interest.

#### Potential concerns

Regardless how awareness is assessed when using CFS, there are several considerations to keep in mind. A concern that can arise in the context of subjective awareness measures is that of decision criterion. Specifically, when asking an observer whether he or she perceives a stimulus, a negative response may reflect a conservative criterion rather than lack of awareness of the stimulus (Eriksen, 1960; Holender, 1986). Although this point applies generally to experiments that measure awareness subjectively, it may be particularly pressing in the case of CFS experiments, given that a stimulus pitted against a dynamic CFS display may partially break suppression but rarely overcomes suppression *completely* such that it achieves exclusive dominance. There exists, in other words, a potentially confusing “gray zone” between seeing nothing and, then, experiencing a clean break from suppression. In line with this notion, accruing evidence demonstrates that visual awareness of complex stimuli does, indeed, vary in a graded fashion, both under visual masking (e.g., Overgaard et al., 2006; Seth et al., 2008; Sandberg et al., 2010) and under CFS (Kang et al., 2011; Mudrik et al., 2013).

The possibility of partial visibility during CFS is an important concern in the context of dichotomous subjective report tasks, given that partially visible stimuli that may not elicit a “yes” response in such a task, are likely to nevertheless affect experimental measures. Visual masking studies have shown that the strength of semantic priming correlates with the degree of perception of prime stimuli (e.g., Purcell et al., 1983; Kouider and Dupoux, 2004; Nolan and Caramazza, 2013; review by Kouider and Dehaene, 2007; see also Kouider et al., 2010). To give an example, Kouider and Dupoux (2004) assessed observers’ awareness using tasks that tapped into different stages of stimulus processing and observed semantic priming by partially visible words in which observers could accurately discriminate letters yet without recognizing the words as a whole. When observers could neither recognize the words nor discriminate letters, indicating that the words were fully masked, semantic priming was completely abolished.

Objective measures of awareness, in turn, are not free from drawbacks either. Both objective and subjective measures have been critiqued on statistical grounds (Rouder et al., 2007; review by Hesselmann, 2013). Specifically, experimenters who use a yes–no, detection, or discrimination task as their index of awareness may find no significant difference between an observer’s objective performance and chance level performance, and then may falsely accept the null hypothesis that observers’ performance is equivalent to chance levels, when in reality the experiment is underpowered to detect a reliable difference (Altman and Bland, 1995). Second, whereas the first point suggests that objective measures can be overly liberal in identifying situations as lacking awareness, objective measures have also been argued to be overly conservative. That is, above-chance performance on discrimination tasks could in some cases be attributed to influences that are not accompanied by phenomenal experience, and that may therefore be classified as outside of awareness (Cheesman and Merikle, 1986; Merikle and Daneman, 1998). In such cases there is, therefore, a dissociation between subjective and objective measures of awareness (Stoerig and Cowey, 1997; Kanai et al., 2010). As we will discuss below, this is certainly not the only dissociation between different measures of awareness.

Several additional concerns arise from the fact, mentioned above, that performance levels on the objective task are usually assessed in a control experiment separately from the main experiment. During such a control experiment, the observer is typically instructed to perform a task on a suppressed stimulus across consecutive trials and in the absence of feedback. If CFS is indeed successful at effectively suppressing the stimulus on a majority of the trials, the observer will fail to detect the stimulus over and over again, and there is evidence that this can lead to an underestimation of the observer’s true performance levels. For example, one study compared detection of a masked stimulus under two conditions (Pratte and Rouder, 2009; see also Lin and Murray, 2014). One condition consisted exclusively of trials involving this masked stimulus, whereas the other condition also included trials where the stimulus was perceptually visible. In this second condition, observers could reliably detect masked stimuli, but in the first condition, detection performance was at chance level for those same stimuli, arguably because an inability to detect the stimulus on a large proportion of the trials caused inattention or lack of motivation. Similar effects of impaired performance have been observed in visual search experiments where only small a minority of trials contains an actual target (Wolfe et al., 2007).

Aside from this issue of inattention or lack of motivation, two other factors can limit the extent to which awareness measures obtained in a control experiment may not generalize to the main experiment. First, given that the two experiments typically involve different behavioral tasks, observers’ strategies are likely to differ in time, potentially leading to differences in awareness (Reingold and Merikle, 1988). Second, perceptual sensitivity and response criteria may vary over time due to adaptation, fatigue or training, disqualifying any techniques that do not allow one to separate this variation from the measure of interest (e.g., Purcell et al., 1983). We should add that these concerns about testing for awareness outside of the main experiment apply with equal force to situations in which awareness measures are obtained from observers different from those tested in the main experiment (Bahrami et al., 2010; Xu et al., 2011; Troiani and Schultz, 2013). Indeed, we see no justification for doing this. The strength of interocular suppression differs considerably among observers; a CFS mask of given contrast may render a dichoptically viewed target completely undetectable for one observer, but for another observer this same CFS mask may prove relatively weak in terms of suppressing a target (Yang et al., 2010a; Zadbood et al., 2011). Despite CFS’s reputation for producing potent suppression, individual differences do exist and they could substantially impact the influence of other factors modulating awareness.

Our final concern about objective awareness measures during CFS relates to the point we raised above when discussing subjective awareness measures, and the concern centers on the possibility of partial visibility. When sensory signals are weak or degraded but, at minimal, detectable, observers may fail to consciously access information at different levels of processing and thus different representational levels (Kouider and Dupoux, 2004; Kouider et al., 2010). In the context of objective measures, an observer’s awareness is commonly indexed by his or her ability to discriminate between two alternatives in a categorization task on the suppressed stimulus. For instance, an observer may be asked to report whether the stimulus is a tool or an animal (Almeida et al., 2008, 2010). Classifying an image into categories such as these plausibly requires more information than does merely detecting the presence of that image. If a stimulus becomes partially visible, therefore, the situation is similar to the one we described above when discussing subjective awareness measures. Specifically, partial visibility may not be sufficient for performing the classification task used to index awareness, but it may nevertheless influence the independent measure (e.g., priming, adaptation) investigated by the study at hand. In support of this notion, Mudrik et al. (2013) demonstrated that a face suppressed with CFS caused priming when using chance performance on a face identification task as the criterion for including data, but this priming disappeared when the authors instead selected data on the basis of a more stringent location discrimination task.

We will conclude this section with the general note that there is probably no single, foolproof index of awareness. For instance, in the case of “blindsight” subjective and objective measures of awareness conflict with one another. Here cortically lesioned patients deny having any subjective awareness of visual stimuli but can successfully perform objective tasks on those stimuli (e.g., Sahraie et al., 1997; Stoerig and Cowey, 1997; de Gelder et al., 2008). There is evidence for similar dissociations in healthy individuals as well (Kolb and Braun, 1995; Lau and Passingham, 2006). Objective and subjective measures of awareness are not only dissociable at the behavioral level, as Hesselmann et al. (2011) have demonstrated. These authors employed functional magnetic resonance imaging (fMRI) while observers made objective and subjective reports of stimuli suppressed with CFS. Areas beyond early visual cortex were strongly responsive to trials in which observers subjectively reported seeing the “suppressed” stimulus, whereas objective performance on a location discrimination task was correlated with multivariate pattern classification performance using responses from early visual areas.

#### Recommendations

Based on the considerations detailed above, we come to the following suggestions for a “best practice” approach to measuring the degree of awareness of the suppressed stimulus in CFS experiments.

It should be clear from the above that each awareness measure has its own shortcomings, and also that different awareness measures plausibly index different stages of awareness. This leads to two recommendations. First, it is reasonable to employ multiple measures of awareness side by side, to obtain a more complete assessment of observers’ perceptual state under CFS (Sterzer et al., 2008; Kang et al., 2011; Kaunitz et al., 2011b; Yokoyama et al., 2013). Second, it is advisable to be cautious in generalizing findings obtained using one criterion of unawareness, to situations that employ other measures.

Emerging from the considerations raised in this section is a common theme: the transition from complete awareness to unawareness is not abrupt but, instead, unfolds in a graded fashion. With regard to subjective measures of awareness, this means that it is advisable to use report scales with multiple levels, rather than dichotomous ones, to obtain more certainty that awareness was, in fact, lacking in conditions labeled as “unaware.” To illustrate this point, studies by Overgaard et al. (2013) have shown that subliminal perceptual effects using a dichotomous measure were no longer observed when executing the same experimental paradigm with the PAS graded report scale. With regard to objective measures, the graded nature of awareness during CFS means that asking observers to cast a verdict about relatively complex stimulus aspects (e.g., semantic category) invites the possibility of overlooking their awareness of basic stimulus features insufficient to perform that task. In other words, it is preferable to ask observers to perform a task on basic stimulus features, instead.

We mentioned the statistical concern that has been raised with regard to objective awareness measures, of falsely accepting the null hypothesis when objective performance does not significantly differ from chance. When aiming to substantiate an unawareness claim using objective measures, it would be a good idea to perform some type of power analysis to minimize a Type II error. For instance, one can use a method involving equivalence confidence intervals (Berger and Hsu, 1996; Overgaard et al., 2013), where one identifies the range of potential values of the dependent variable that would be statistically indistinguishable from chance performance, and then evaluates whether both the lower and upper confidence intervals around the observed variable lie within this range.

As a final recommendation, we pointed out concerns that arise when measuring awareness in an experiment separate from the main one, and the best way to sidestep those concerns is to include the awareness condition within the main experiment. A good example of such an approach was offered by Faivre et al. (2012). Within a single experiment, these authors randomly intermixed trials that required an awareness judgment and ones where the main task was required. Observers were not informed until the end of a trial which kind of trial it was, thus guaranteeing a similar attitude in terms of attention and motivation across both trial types. In situations where it is infeasible to obtain visibility measures within the main experiment, it is useful to at least employ a maximally similar paradigm and task set across both experiments (Reingold and Merikle, 1988) and, ideally, to assess awareness both prior to the main experiment and afterward (e.g., see Jiang and He, 2006; Kang et al., 2011).

### WHAT ARE EFFECTIVE METHODS FOR GAGING THE SPECIFICITY AND STRENGTH OF STIMULUS PROCESSING OUTSIDE OF AWARENESS?

A common approach when investigating stimulus processing outside of awareness with CFS is to contrast a given measure in the presence of interocular suppression against that measure in absence of interocular suppression. In priming and adaptation paradigms, these measures tend to map onto conditions where stimuli are visible or invisible owing to CFS. In b-CFS, these measures are typically represented by conditions in which stimuli gradually become visible as a result of either emergence from suppression or some type of stimulus manipulation such as contrast ramping. The comparison of stimulus-driven effects in the presence and absence of interocular suppression allows investigators to index the relative strength and specificity of stimulus processes engaged without an observer’s awareness. Take binocular rivalry, for instance, where one can directly compare a stimulus-driven effect when that stimulus is perceptually dominant as opposed to when it is suppressed during rivalry while all other aspects of the stimuli and procedures remain unchanged. This particular approach of holding all conditions identical with the sole exception of an observer’s awareness is rather difficult when using CFS, because stimuli are so infrequently and incompletely perceived in the presence of a potent CFS mask. Thus, here CFS’s extreme effectiveness for rendering stimuli invisible becomes, paradoxically, a potential drawback. Indeed, this may explain why so many CFS studies that use a priming paradigm choose not to include a visible condition at all: priming effects known to exist based on previous research using visible stimuli were only assessed using prime stimuli rendered invisible by CFS (Almeida et al., 2008, 2010, 2013; Anderson et al., 2012; Faivre et al., 2012; Sakuraba et al., 2012; Zabelina et al., 2013). In these studies, evidence for priming despite CFS is revealing, particularly when the strength of these subliminal priming effects varies across stimulus categories (e.g., tools versus faces). Still, it remains unclear whether priming without awareness is different in magnitude relative to priming with visible stimuli.

Many other CFS studies with priming and adaptation paradigms do include a no-suppression condition, but the potency of CFS often forces researchers to specifically design that condition rather than simply wait for the target stimulus to break through suppression and become visible (for exceptions see Adams et al., 2010; Stein and Sterzer, 2011; Stein et al., 2012a). Next we turn to such CFS studies that do include a no-suppression condition. We will dedicate a separate section of this discussion to paradigms that involve the target stimulus b-CFS, because the choice of the no-suppression condition turns out to be particularly important in those paradigms.

#### General concerns regarding no-suppression conditions in CFS paradigms

For the no-suppression condition of many physiological and behavioral studies, the CFS mask was simply removed altogether, thereby leaving the monocular stimulus viewed by the other eye easily visible (e.g., Kanai et al., 2006; Yang et al., 2010b; Amihai et al., 2011; Sweeny et al., 2011; Sklar et al., 2012). One potential drawback to this kind of monocular condition is that measures of stimulus-driven effects with the monocularly visible stimulus may be inflated, since removing the CFS mask may also eliminate a large source of external noise. Moreover, removal of the CFS mask may well influence the extent to which contrast normalization influences the effective contrast of the combined left- and right-eye neural signals independent of interocular suppression (e.g., Ding and Sperling, 2006; Said and Heeger, 2013). Consistent with these concerns, some pivotal conclusions based on fMRI results obtained using CFS in combination with a no-mask comparison condition (Fang and He, 2005; Jiang and He, 2006; Sterzer et al., 2008; Vizueta et al., 2011) were not reached in studies where CFS masks were included and matched across experimental conditions (Hesselmann and Malach, 2011; Hesselmann et al., 2011).

#### No-suppression conditions in b-CFS paradigms

As briefly discussed above, the b-CFS paradigm, unlike other paradigms used with CFS, aims to infer processing of a target stimulus that is suppressed from awareness, by measuring at which moment this suppression ends and the observer becomes aware of that same stimulus (e.g., Jiang et al., 2007; Stein et al., 2011b). In other words, the paradigm, by design, operates right on the border between awareness and unawareness. The same is not true for paradigms involving, say, adaptation or priming, which index processing outside of awareness in terms of detection or performance in a separate task. Just to review, the logic of the b-CFS paradigm entails comparing the RT at which an initially suppressed target stimulus is first detected as it emerges from CFS. When different classes of stimuli show significantly different detection RTs, one presumes that those stimulus categories were differentially processed while suppressed, with faster RTs implying more robust processing outside of awareness. For instance, emotional faces are detected faster than their neutral counterparts as they emerge from CFS (Yang et al., 2007; Tsuchiya et al., 2009; Gray et al., 2010; Sterzer et al., 2011; Stein and Sterzer, 2012; Stein et al., 2014). However, given the characteristic of the b-CFS paradigm of relying on responses made when the target stimulus is *not* suppressed, one needs to be cautious ascribing RT differences to differences in processing outside of awareness. For instance, RT may also be modulated by factors such as general detection ability, response criteria, and basic visual attributes. Ruling out such alternative explanations means that the choice of no-suppression condition is critical, as detailed below.

Alternative explanations to processing outside of awareness can be rejected by demonstrating that RT differences found between two stimulus conditions using the b-CFS procedure disappear when interocular suppression is removed from the picture. The no-suppression condition that is required to demonstrate this should ideally engage all the processes that occur in the invisible condition, with the exception of those that render stimuli invisible (Stein et al., 2011b). In one popular no-suppression condition, the target stimulus is blended into the CFS mask itself so that both target and CFS mask are seen by the same eye, rather than having the target imaged in the eye not viewing the CFS mask, as in the invisible condition (e.g., Costello et al., 2009; Yang and Yeh, 2010; Mudrik et al., 2011; Stein and Sterzer, 2012; Stewart et al., 2012). The blending is implemented so that the target stimulus gradually emerges within the CFS mask during the trial, to perceptually mimic that stimulus emerging from suppression (but see Stein et al., 2011b). As we will discuss next, this approach comes with several subtleties and potential pitfalls.

A primary concern with this kind of “blended” condition is that the resulting RT distribution is almost always significantly different from the RT distribution found for the invisible condition. Most notably, in the no-suppression condition RTs are typically faster and less variable than they are in the invisible condition. The reduced variability, in particular, can be a cause for concern. For instance, a small RT range suggests that the target stimuli may be transitioning from unnoticeable to noticeable rather abruptly. As a result, even if observers do reach decisions about the presence of different stimulus categories at different rates in the blended condition, those differences may be obscured by floor effects that mask decisional influences (e.g., response criterion), which are more evident in the longer RTs measured in the invisible condition.

Another aspect of the relatively small RT variability in the no-suppression condition is related to the fact that the ramping rate typically remains fixed across trials. This constant rate means that observers can develop strong expectancy effects for this condition, effects that are precluded in the invisible conditions by the temporal uncertainty engendered by the more variable durations of suppression. Any differences in expectation between the no-suppression condition and the invisible condition may also lead to differences in observer’s response strategies, and these differences are further exacerbated when no-suppression and invisible trials are presented in separate blocks, as often done in CFS studies (Stein et al., 2011b).

#### Recommendations

For certain CFS designs involving measures such as adaptation and priming, the concerns expressed above make it unwise to compare CFS conditions to conditions without any mask whatsoever. If the CFS masks are truly potent, only a small percentage of trials will fail to produce reliable suppression. It is then possible, with some adaptation paradigms, to compare trials with CFS masks in which suppression succeeded to those in which suppression failed (Adams et al., 2010; Stein and Sterzer, 2011; Stein et al., 2012a). One potential drawback is that it may be laborious to acquire a sufficient number of no-suppression trials. Alternatively, one should try to tone down the CFS mask to allow periods where the target stimulus becomes unequivocally visible. Then, periods of suppression and periods of visibility can be compared, all in the presence of a CFS mask. Some studies have taken steps in this direction, and measured the stimulus conditions that produced reliable suppression for each participant, prior to the main task. In doing so, those studies were able to distinguish stimulus conditions (e.g., contrast values) that different levels of stimulus awareness in the presence of the CFS mask (e.g., Tsuchiya and Koch, 2005; Hesselmann et al., 2011; Kang et al., 2011; Kaunitz et al., 2011b). While more time consuming, measuring CFS under these graded visibility conditions provides a more complete picture of the impact of CFS and visibility. Moreover, conditions with different degrees of visibility in this design may also be more comparable in terms of detectability and attentional engagement.

As for the b-CFS paradigm, there are several improvements in the no-suppression condition that could be implemented. To minimize differences in RT distributions across no-suppression and invisible conditions, the rate at which a stimulus increases in luminance or contrast during a no-suppression trial can be varied to produce RT distributions similar to those measured in the invisible condition (e.g., Stein et al., 2011b, 2012b). After successfully matching RT distributions across conditions with this and related methods, Stein et al. (2011b) found that one differential stimulus effect – the face inversion effect – produced under the invisible condition was observed in the no-suppression condition as well.

To circumvent effects attributed to differences in anticipation and response strategies, it is advisable for no-suppression and invisible trials to be randomly intermixed and, when possible, for response accuracy to be measured rather than RT (Stein et al., 2011b). In fact, this suggestion is applicable to all techniques using CFS. In addition to intermixing no-suppression and invisible trials, varying stimulus onset in both no-suppression and invisible conditions can further reduce differences in temporal uncertainty and minimize anticipation effects in no-suppression trials (Sterzer et al., 2011; Stein and Sterzer, 2012; Gayet et al., 2013). Explicitly modeling response bias or decision criterion (e.g., diffusion models by Smith and Ratcliff, 2004) can at least dissociate RT effects driven by bias in stimulus processing from those driven by more post-perceptual, cognitive (e.g., decision making) processes.

Putting aside, for now, the methodological challenges associated with designing a no-suppression condition for b-CFS, we would like to conclude with a conceptual point. Many studies using b-CFS have now reported stimulus factors that influence the time it takes to report an initially suppressed stimulus. Indeed, such effects have been reported for a broad range of stimulus categories, including words, scenes and faces. True to the logic of no-suppression conditions outlined above, these same studies did not find the same effects on RT when the stimuli were not suppressed, indicating the specific involvement of processes outside of awareness. This remarkable situation raises the question why preferential processing should occur only in the absence of awareness, and why the same mechanisms that affect processing outside of awareness would not influence conscious processing as well. One possibility is that similar influences do affect conscious processing as well, but that these influences can remain undetected for methodological reasons such as the ones outlined above. For instance, early studies have reported shorter RTs for upright faces than for inverted faces during b-CFS but not during visible control conditions, yet more recent work has shown similar effects for visible conditions as well (Stein et al., 2011b). The more fundamental question, however, is this: what kind of processes are we left with when comparing a b-CFS condition with an ideal control condition that is matched in everything but interocular suppression?

As a final methodological suggestion for all CFS paradigms we would like to point to an approach in the literature that has not yet been used in combination with CFS, but whose properties may enable this approach to circumvent some concerns associated with no-suppression conditions. This type of approach, traditionally known as the process-dissociation method, capitalizes on experimental measures that differ qualitatively in awareness (Marcel, 1983; Cheesman and Merikle, 1986; Jacoby, 1991). This relies on the notion that awareness allows observers to intentionally act on information provided by the stimulus, yet that the absence of awareness leads to more automatic reactions to stimulus information that observers cannot intentionally control (Merikle et al., 2001). In a classic example (Debner and Jacoby, 1994; Merikle et al., 1995), an image of a word is perceptually masked and then followed by an image containing the first three letters of that same word. Observers’ instructions are to complete the word stem with the first word that came to mind excluding the word that was previously presented. If the previous word was effectively masked, observers are more likely to recall that word when filling in the word stem (i.e., priming effect). However, if the word was visible to observers, they should be able to prevent themselves from using that word. While some concerns have been raised with this particular example (e.g., Fisk and Haase, 2007), there are several other circumstances that can generate qualitatively different effects based on stimulus visibility (e.g., Murphy and Zajonc, 1993; Merikle and Joordens, 1997).

### WHAT ARE FACTORS THAT INFLUENCE THE ROBUSTNESS OF STIMULUS-DRIVEN EFFECTS UNDER CFS?

#### Attention

While the relationship between attention and awareness remains controversial, there is growing consensus that that attention and awareness can to some extent be dissociated (reviews by Koch and Tsuchiya, 2007; van Boxtel et al., 2010a; but Cohen et al., 2012). For instance, lines of research that we will discuss below, suggest that (1) attention can be involuntarily drawn to the location of a stimulus suppressed by CFS, and (2) that attention voluntarily directed toward the location or features of a suppressed stimulus can significantly diminish the extent to which processing of that stimulus is impacted by CFS suppression. While both these notions indicate that CFS suppression and inattention can be separated, the second notion suggests something else as well. Specifically, under the reasonable assumption that observers in standard CFS designs are inclined to pay less attention to the location and features of a target stimulus once they can no longer see the stimulus due to suppression, this second notion leads us to ask whether the reduction in neural processing that is often observed for a suppressed stimulus may, in part, be due to lack of attention to that stimulus. Incidentally, a related issue has been the center of a long-standing debate involving affective priming by stimuli rendered invisible using backward masking (reviews by Pessoa, 2005; Bishop, 2008). Early studies reported subliminal priming effects with and neural responses to affective stimuli rendered invisible with backward masking, pointing to affective processing outside of awareness (e.g., Morris et al., 1998; Whalen et al., 2004). However, recent studies have demonstrated that these effects disappear when attention is sufficiently allocated away from affective stimuli, suggesting that affective processing outside of awareness is conditional upon attention mechanisms (e.g., Phillips et al., 2004; Kouider et al., 2009). In the following section, we will discuss studies of attention manipulations during CFS, as well as their relevance for work examining visual processing under CFS suppression.

First, attention can be involuntarily drawn to the location of a stimulus suppressed by CFS. Specifically, certain categories of stimuli, such as arousing images (Jiang et al., 2006) and emotional facial expressions (Yang et al., 2011), have been shown to attract observers’ attention toward the location of those stimuli, even when they are suppressed from awareness with CFS. As a consequence, these invisible stimuli either facilitate or hinder observers’ responses to subsequent visible stimuli, which are presented in corresponding or opposing spatial locations. In a related finding, a search task involving a target that was suppressed using CFS revealed that the eyes fixated longer on the location of the target, even though it remained unperceived (Rothkirch et al., 2012). There is also evidence that stimuli such as suppressed faces with averted gaze can cue observers’ endogenous spatial attention (Xu et al., 2011). Interestingly, similar findings have not been reported with binocular rivalry, as suppressed visual cues failed to influence observers’ spatial attention in a related design (Schall et al., 1993).

Second, attention voluntarily directed to an invisible stimulus can strongly increase the extent to which that stimulus is processed outside of awareness. Specifically, when an observer’s spatial or feature-based attention is purposely directed at an invisible stimulus, the effective strength of that visual stimulus is enhanced, as evidenced by the stronger visual aftereffects it induces despite its invisibility (Kanai et al., 2006; Shin et al., 2009; Yang et al., 2010b). Conversely, these aftereffects are substantially weakened, if not abolished, if attention is purposely removed from the suppressed stimulus (Bahrami et al., 2008; Shin et al., 2009; Kaunitz et al., 2011a). In other words, directing attention to the location or to the features of an invisible stimulus modulates the degree to which that stimulus is processed outside of awareness. Physiological support for this notion comes from studies showing that attention allocation and attentional load directly modulate fMRI BOLD responses to stimuli suppressed from awareness by CFS (Bahrami et al., 2007; Watanabe et al., 2011; Yuval-Greenberg and Heeger, 2013). One possible role of attention outside of awareness is to temporarily bind the encoded features of an invisible stimulus to create high-level representations that guide behavioral and perceptual processes outside of awareness (Lin and He, 2009).

In summary, attention may modulate the extent of visual processing under suppression, successfully boosting or weakening neural signals arising from the suppressed stimulus. Considering that inattention to a stimulus is a common, but apparently not necessary, concomitant of perceptually suppressing that stimulus, this observation is important for CFS studies that report reduced or abolished stimulus-driven effects under CFS. Rather than concluding that those effects are modulated by awareness alone, one needs to consider the possibility that they are, at least in part, modulated by attention. To give an illustrative example, one topic that calls for such a cautious attitude is the topic of high-level aftereffects induced by stimuli under CFS. Both complex motion aftereffects (Maruya et al., 2008; Kaunitz et al., 2011a) and various face aftereffects (Shin et al., 2009; Yang et al., 2010b; Amihai et al., 2011; Stein and Sterzer, 2011) can be fully erased by CFS suppression of the inducing stimulus, yet this can to some extent be prevented by making sure the observer keeps attending to the location of the suppressed stimulus (Shin et al., 2009; Yang et al., 2010b; Kaunitz et al., 2011a).

#### Feature-selective suppression

Many studies of binocular rivalry show that interocular suppression adversely impacts visibility for a broad range of stimuli presented under suppression. Nearly all rivalry studies involving detection or discrimination of test probes have shown that an observer’s ability to perceive a wide range of visual features is significantly impaired when probes are presented during interocular suppression, including probes that differ greatly from the currently suppressed target (review by Blake, 2001). It was that pattern of results that led to the characterization of interocular rivalry suppression as non-selective, meaning all classes of visual input are affected, to some extent, when presented under suppression (review by Blake and Logothetis, 2002). This view of suppression dovetails with other findings suggesting that interocular suppression works by reducing effective stimulus contrast or contrast gain of stimulus-evoked responses within early stages of visual processing (Watanabe et al., 2004; Tsuchiya et al., 2006; Yuval-Greenberg and Heeger, 2013). However, this is only half of the story, for there is also evidence for an additional selective component to interocular suppression (Stuit et al., 2009). In the following paragraphs, we review evidence for selectivity obtained using the CFS technique to induce interocular suppression.

Several lines of evidence suggest that processing of some classes of stimuli is less adversely impacted by suppression than is processing of others when CFS is used to induce interocular suppression. For instance, neural processing of objects that fall within categories such as tools and emotional faces appears less susceptible to CFS than is neural processing of stimuli in animal, vehicle, and neutral face categories, as evidenced by the stronger stimulus-induced effects produced by tools and emotional faces under suppression. In this sense, interocular suppression produced by CFS selectively attenuates or abolishes certain signals while leaving others to be potentially encoded during suppression. However, the underlying mechanism for such selective suppression is unclear. Lin and He (2009) proposed a framework in which some critical stimulus features can be registered under CFS, with the degree to which this happens dependent on the type of visual input. Specifically, suppression, in this view, is strongest within functionally specialized areas that comprise the ventral visual pathway, areas in which activity is thought to correlate strongly with object representations (e.g., Rees et al., 2002). In contrast, areas that are relatively unperturbed by CFS may be those comprising the dorsal visual pathway (Fang and He, 2005), as well as the subcortical pathways, presumably more primitive neural circuitry in evolutionary terms and responsible for registering ecologically relevant information including affective content (Morris et al., 1998; Jiang and He, 2006). Lin and He’s view is consistent with popular theories supporting the functional significance of dorsal visual and subcortical affective pathways in guiding behavior outside of awareness (e.g., Goodale et al., 1991; Ohman and Mineka, 2001). At the same time, however, evidence consistent with this dorsal/ventral distinction has been challenged by several recent studies demonstrating the importance of feature-based encoding during CFS. These studies are discussed below.

The apparent dissociation in dorsal and ventral stream processing under CFS is primarily supported by reports of object-selective processing of tool images, which are presumably registered within the dorsal stream (Fang and He, 2005; Almeida et al., 2008, 2010). Almeida et al. (2008, 2010) first showed category-related priming effects that were specific to images of tools. However, those investigators did not take into account that, unlike other object categories tested (i.e., animals), tools tend to be elongated in shape, and Sakuraba et al. (2012) later demonstrated that this may be an important factor. Specifically, these latter authors argued that tool-selective priming with CFS was more likely attributable to the encoding of object shape rather than object category, based on their finding that elongated non-tool objects elicited equivalent priming effects whereas non-elongated tools failed to produce any priming (see also Kaunitz et al., 2011b). In addition, physiological evidence for preferential encoding of tools in dorsal areas under CFS (Fang and He, 2005) has not been replicated when CFS displays were presented in both visible and invisible conditions (Hesselmann and Malach, 2011; Hesselmann et al., 2011). Exclusion of the CFS mask in the visible condition may make it difficult to dissociate responses linked to differences in stimulus awareness and those related to discrepancies in stimulus conditions.

The subcortical hypothesis for emotion processing posits that threat-related stimuli are prioritized during stimulus processing that may occur pre-attentively and outside of awareness (review by Phelps and LeDoux, 2005). This theory has been supported by CFS studies demonstrating that fearful face stimuli evoke greater neural responses and break suppression faster than other emotional and neutral face stimuli during CFS (Jiang and He, 2006; Yang et al., 2007; Jiang et al., 2009; Sterzer et al., 2011; Gray et al., 2013; Troiani and Schultz, 2013; Stein et al., 2014). However, a series of recent behavioral studies have shown that this advantage in breaking suppression may not be attributed to the emotional content of fearful faces and may not specifically involve the subcortical pathway. For instance, Gray et al. (2013) demonstrated a similar advantage in breaking suppression for face stimuli that were identical in several low-level visual properties as fearful faces (i.e., spatial frequency, contrast) but were not explicitly or implicitly recognized as fearful in expression. This study thus suggests that the rapid detection of fearful faces may be attributed to properties other than emotional content. In addition, Stein et al. (2014) showed that this fear-based advantage is modulated by differences in high rather than low spatial frequency content across emotional expressions (see also Stein and Sterzer, 2012), which is consistent with a recent study showing that high spatial frequency content is less susceptible to CFS suppression than low spatial frequency content (Yang and Blake, 2012). The Stein et al. (2014) study does not implicate the subcortical pathway since it is thought that this route predominantly conveys coarse low spatial frequency information of threat-related stimuli to the amygdala (Vuilleumier et al., 2003). An additional piece of evidence against the involvement of the amygdala is that patients with bilateral or unilateral lesions to the amygdala show an intact fearful face advantage during CFS (Tsuchiya et al., 2009; Yang et al., 2012; see also Willenbockel et al., 2012).

In summary, differential processing of low-level features likely played a larger role than originally anticipated in several studies using CFS, contributing to the impression that particular routes of visual processing were relatively unaffected by CFS.

***Differential suppression of low-level features.*** Several studies mentioned above underscore the influence of low-level stimulus properties in stimulus-driven effects obtained under CFS suppression. In this context it is important to note that interocular suppression may differentially affect the encoding of different low-level features. Specifically, studies using binocular rivalry (e.g., Yang et al., 1992; Alais and Parker, 2006; Alais and Melcher, 2007), dichoptic masking (e.g., Baker and Meese, 2007) and more recently CFS (Hong and Blake, 2009; Zadbood et al., 2011; Yang and Blake, 2012) have demonstrated that stimulus features most strongly suppressed are those that are shared with the stimulus that induces suppression, or the “suppressor.” Recent work shows that this general pattern also applies to the Mondrian-like CFS display that was introduced by Tsuchiya and Koch (2005), and that is the most popular version of CFS display currently in use. Yang and Blake (2012) demonstrated that the features most strongly suppressed by Mondrian patterns are low spatial frequencies and cardinal orientations, which also happen to be the most prominent features of the Mondrian patterns themselves (see also Tsuchiya and Koch, 2005). Furthermore, altering the spatiotemporal properties of the Mondrian patterns also altered the pattern of suppression, such that stimulus features shared by the suppressor were nearly always the ones most strongly suppressed. Stein et al. (2014) found a similar pattern of results with face stimuli and CFS patterns that were varied in spatial frequency content. These investigators also examined suppression with CFS displays that were equivalent in energy across low and high spatial frequency bands. However, suppression remained biased toward low spatial frequency faces even with these filtered displays, and this may be partly attributable to the temporal structure of CFS displays (Yang and Blake, 2012). Other characteristics of CFS using Mondrian displays, include differential suppression of chromatic and achromatic content (Hong and Blake, 2009) and differential suppression of temporal and form information (Zadbood et al., 2011). Finally, the pattern of feature-selective suppression demonstrated with the Mondrian display may generalize to other CFS displays previously used, since Yang and Blake showed that these tend to have spectral profiles similar to that of the Mondrian display (Supplementary Figure 1 in Yang and Blake, 2012). It may be this particular spectral profile that leads to the potent suppression evoked by CFS.

Considered together, the processing of a stimulus under CFS will be adversely impacted in general (non-selective suppression) but to an extent that depends on the similarity between that stimulus and the stimulus doing the suppression [feature-selective suppression, similar to that described by Stuit et al. (2009), for conventional binocular rivalry]. This is important for at least two reasons. First, weakly suppressed stimulus features are more likely to be visible to observers but experimenters may fail to detect observers’ awareness of them (see section above). As a result, visible stimulus fragments may modulate stimulus-driven effects that are mistakenly attributed to processing outside of awareness. Second, even for fully suppressed stimuli, information processing under CFS is determined, in part, by differences in the extent to which basic visual features are impacted by CFS. These two notions are important for studies in which stimulus-driven effects are attributed to encoding of high-level, semantic information during CFS (review by Lin and He, 2009), and in particular when experimenters use stimuli whose similarity in basic visual features is larger within the same semantic category than it is between categories (Kouider and Dehaene, 2007). Especially when selective feature encoding is paired with factors such as small stimulus sets and high rates of stimulus repetition across trials, feature-selective suppression may play a large role in stimulus-driven effects under CFS. In sum, the differential impact of CFS on low-level stimulus features must be considered as an alternative explanation for findings that might otherwise be attributed to high-level visual processing under CFS. Conversely, one may get some idea of the extent to which various later stages of analysis could still function under CFS by considering the extent to which CFS selectively inhibits the basic visual signals that provide input to those stages.

#### General recommendations

We recommend that observers’ attentional state be carefully controlled during tasks involving CFS. For example, attention can be cued to the location of the suppressed stimulus to maximize visual processing under suppression. Importantly, when select visual processes are hypothesized as being engaged automatically or in the absence of awareness, it should be made explicit whether these processes are also independent of observers’ attentional engagement. One common approach to examining the role of attention in visual processing outside of awareness is to compare the strength of stimulus-driven effects under conditions where attention is directed toward versus away from suppressed target stimuli.

To avoid the potential effects of feature-selective suppression described above, we recommend that experimenters select target images that are similar in spatial composition (e.g., shape, size) within and across stimulus categories or, better yet, create images comprised of different phase spectra but identical amplitude spectra using image processing techniques. Secondly, certain spatial properties can be normalized across stimuli such as spatial frequency amplitude, contrast, mean luminance, orientation content, shape, and size. Finally, we recommend that experimenters use CFS displays that are similar in spatial profile as the target stimuli to be suppressed, in order to maximize suppression of all components of the target stimulus. Alternatively, one can manipulate stimuli to have similar spatial profiles as the CFS display without necessarily altering stimulus recognition. Based on previous studies (Hong and Blake, 2009; Yang and Blake, 2012), achromatic Mondrian displays may prove to be most effective at suppressing static, achromatic images composed of low spatial frequency, cardinally oriented features. Considering that visible or weakly suppressed features may still occur, one further measure one can take is to use large stimulus sets or to replicate findings with multiple stimulus sets, to minimize learned associations or effects of stimulus repetition across trials.

## CONCLUSION

Those of us interested in visual processing outside of awareness have at our disposal an impressive array of tools for manipulating visual awareness. Among those tools, CFS is particularly appealing, since it offers several advantages compared to other techniques at rendering stimuli invisible. Not surprisingly, the technique has caught on within the field, and the volume of results for processing outside of awareness obtained with this technique is already quite substantial despite the technique’s appearance on the scene <10 years ago. Surveying studies that have employed CFS, one sees several different ways in which CFS is being exploited, each with its own subtleties that may influence the likelihood of finding evidence for visual processing outside of awareness. Researchers intending to use CFS will learn that multiple factors must be taken into consideration when using this technique; we believe the majority of those considerations can be grouped into four primary questions. In this paper, we have provided recommendations for addressing those questions, and those recommendations are briefly reiterated below.

(1) What are suitable paradigms to use with CFS to study processing outside of awareness?
  • Measures of adaptation aftereffects are useful in examining neural processes involved in the encoding of visual attributes of a stimulus.
  • Priming paradigms allow researchers to examine processing of physical and conceptual (semantic) characteristics shared by two stimuli.
  • The b-CFS paradigm relies on the speed at which stimuli emerge from suppression to infer the relative strength of stimulus processing outside of awareness. This technique has been widely used in examining semantic processes outside of awareness.
(2) What are the optimal ways to determine whether a stimulus is genuinely invisible?
  • To obtain a more complete evaluation of observers’ perceptual state under CFS with adaptation or priming paradigms, we advise the employment of multiple measures of awareness, which include measures that assess different states of awareness (i.e., objective, subjective) and measures that gage different stages of stimulus analysis.
  • We recommend the use of statistical analyses that reduce the likelihood of falsely accepting the null hypothesis that observers’ performance on an awareness measure is not significantly different from chance, with the implication that stimuli were sufficiently rendered invisible.
  • We urge that awareness measures be implemented *within* the main experiment, and that those measures be administered in ways that recreate, as nearly as possible, the attentional state and response strategies engendered during the main task when the target stimulus is putatively suppressed from awareness by CFS.
(3) What are effective methods for gaging the specificity and strength of stimulus processing outside of awareness?
  • Studies should strive to compare stimulus-driven effects found with CFS to those measured without suppression.
  • The no-suppression condition(s) should be matched as closely as possible to the invisible condition.
  • We recommend that no-suppression and invisible trials be randomly intermixed to minimize potential differences between conditions (i.e., differences such as anticipation and response strategy).
  • For the b-CFS procedure, the no-suppression condition should be individually tailored for each participant to produce similar RT distributions in the behavioral task and similar perceptual experiences for the no-suppression and invisible conditions.
(4) What are factors that influence the robustness of stimulus-driven effects under CFS?
  • The spatial location of an observer’s attentional engagement can modulate visual processing under CFS. Thus:
    ◦ It is advisable to hold an observer’s spatial attention constant across trials, particularly directing attention to the location of the stimulus being suppressed so as to maximize the likelihood of stimulus processing under CFS.
    ◦ Researchers should also consider manipulating an observers’ spatial attention to test whether stimulus processing is engaged without awareness *and* without attention.
  • Low-level features of target stimulus and CFS display can modulate the strength and selectivity of suppression.
    ◦ Target images should be closely matched in spatial composition with one another and with the CFS mask.

In closing, we are excited about the future opportunities for learning more about stimulus processing outside of awareness, and we are confident that CFS can provide one effective means for pursuing that question. Coincidentally, the recent resurgence of interest in processing outside of awareness coincides with the forthcoming one-hundredth anniversary of the publication of one of Sigmund Freud’s most famous essays, *The Unconscious* (Freud, 1915). For decades, Freud’s ideas have been construed as quaint but outmoded, relying as they did on anecdote and scientifically untestable conjecture. It is fair to say that Freud’s ideas about the unconscious provided enjoyable literature but fell outside of the domain of serious psychological science. Ironically, we now find ourselves armed with modern techniques like CFS for probing the unconscious, and there appears to be a growing army of troops enlisting to do just that. Our modest hope is that the concerns about CFS and possible solutions we have voiced in this essay will provide useful guidelines for strengthening the inferential potential of CFS. At the same time, we believe that CFS alone is not going to provide a definitive answer to the question of processing outside of awareness. Instead, we will need to use CFS in conjunction with other techniques for manipulating awareness (Kim and Blake, 2005) to arrive at conclusions about stimulus processing outside of awareness that are not method-specific.

## Conflict of Interest Statement

The authors declare that the research was conducted in the absence of any commercial or financial relationships that could be construed as a potential conflict of interest.

## Footnotes

1 We prefer the phrase “processing outside of awareness” to “unconscious processing” because the latter could be confused to imply that we are focusing on neural correlates of consciousness. Our focus is exclusively on the extent to which a visual stimulus remains effective when it is erased temporarily from visual awareness.
